# Supplementary material for: A qualitative exploration of the impact of the COVID-19 pandemic on gender-based violence against women living with HIV or tuberculosis in Timor Leste
Source: PLoS One. 2024 Aug 12;19(8):e0306106. doi: 10.1371/journal.pone.0306106 (PMC11318865; doi:10.1371/journal.pone.0306106)
Supplement: S1 File — (DOCX) [file pone.0306106.s001.docx]

**TETUM INTERVIEW GUIDE**

| **Identidade Partisipante Intervista** | |
| --- | --- |
| Sexu |  |
| Idade |  |
| Nivel Edukasaun |  |
| Profisaun |  |
| Hela Fatin |  |
| Loron no Data Intervista |  |
| Intervista : Perguntas & Resposta | |
| Perguntas 1 Violensia Fisiku  Durante pandemia COVID 19 iha tinan 2020-2021, Tia/Mana/Alin sira hetan violensia fisiku ruma husi familia uma laran? | |
| Bele cerita ita nia pengalaman violensia fisiku hosi familia sia iha uma laran?  Alasan sa mak nalo familia sia halo violensia fisiku ba o? |  |
| Oinsa Tia/ Mana/ Alin Hasoru no Adapta ho situasaun sira ne’e? |  |
| Perguntas 2 Violensia Verbal no diskursu odio (komunikasaun  Durante pandemia COVID 19 iha tinan 2020-2021, Tia/Mana/Alin sira hetan violensia fisiku ruma husi familia uma laran? (Peskizador temi violensia verbal ka liafuan ida-idak iha kraik ba respondent). | |
| Nunebe Tia/Mana/Alin sira hetan violensia sira ne’e?  Tanba sa mak nalo familia sia halo violensia ne’e sia ba o? |  |
| Oinsa Tia/Mana/Alin hasoru noadapta ho situasaun sira ne’e? |  |
| Perguntas 3 Violensia Psikolojia  Durante pandemia COVID 19 iha tinan 2020-2021, Tia/Mana/Alin sira hetan violensia fisiku ruma husi familia uma laran? (Peskizador temi violensia Psikolojia ka ida-idak iha kraik ba respondent). | |
| Nunebe Tia/Mana/Alin sira hetan violensia sira ne’e?  Tanba sa mak nalo familia sia halo violensia ne’e sia ba o? | *Hanesan iha leten* |
| Oinsa Tia/Mana/Alin hasoru no adapta situasaun sira ne’e ? | Ita ema ne matan mos ona mak ita foin koalia diak. Kuandu lanu ne ema nian mak los no ema la rona ita ne. |
| Perguntas 4 Violensia Seksual  Durante pandemia COVID 19 iha tinan 2020-2021, Tia/Mana/Alin sira hetan violensia seksual ruma husi familia uma laran? (Peskizador temi violensia seksual ka ida-idak iha kraik ba responden). | |
| Obriga halo relasaun seksual ne’ebe mak imi la hakarak | . |
| Obriga halo relasaun seksual penetrasaun anal |  |
| Obriga halo relasaun seksual penetrasaun oral |  |
| Seluk-seluk tan (bele aumenta husi responde |  |
| Nunebe Tia/Mana/Alin sira hetan violensia sira ne’e?  Tanba sa mak nalo familia sia halo violensia ne’e sia ba o? |  |
| Oinsa Tia/Mana/Alin hasoru no adapta situasaun sira ne’e ? |  |
| Perguntas 5 Violensia Sosio-Ekonomio  Durante pandemia COVID 19 iha tinan 2020-2021, Tia/Mana/Alin sira hetan violensia Sosio-Ekonomio ruma husi familia uma laran? (Peskizador temi violensia Sosio-Ekonomio ka ida-idak iha kraik ba responden). | |
| 1. Foti ka hadau tia ema nia redimentu |  |
| 1. La autoriza ita servisu hodi hetan salario ruma (hanesan koloka deit ita nia moris hanesan dona de casa) |  |
| 1. Halo asaun ruma hasoru ita, nune’e ikus mai ita labele halo servisu ruma |  |
| 1. La entrega osan ba ita hodi maneja (karik ita hanesan inan) |  |
| 1. Seluk-seluk tan (bele aumenta husi responde) |  |
| Nunebe Tia/Mana/Alin sira hetan violensia sira ne’e?  Tanba sa mak nalo familia sia halo violensia ne’e sia ba o? |  |
| Oinsa Tia/Mana/Alin hasoru no adapta situasaun sira ne’e ? |  |
| Perguntas 6 Violensia Domistiku ka Violensia fen ho laen (Parseiru)  Durante pandemia COVID 19 iha tinan 2020-2021, Tia/Mana/Alin sira hetan violensia seksual ruma husi familia uma laran? (Peskizador temi violensia Domistiku ka Violensia fen ho laen? ka ida-idak iha kraik ba responden). | |
| 1. Violensia fiziku (baku, tuku no tebe) husi laen |  |
| 1. Violensia fiziku no torturasaun husi laen kauza to’o tohar alezadu |  |
| 1. Violensia psikolojia no ekonomia husi laen kauza ita hetan sofrimentu fuan ho laran |  |
| 1. Obriga ita halo relasaun seksual maske ita lakohi |  |
| 1. Seluk-seluk tan (bele aumenta husi responde) |  |
| Nunebe Tia/Mana/Alin sira hetan violensia sira ne’e?  Tanba sa mak nalo familia sia halo violensia ne’e sia ba o? |  |
| Oinsa Tia/Mana/Alin hasoru no adapta situasaun sira ne’e ? |  |
| Perguntas 7 Violensia no Abuzu Seksual  Durante pandemia COVID 19 iha tinan 2020-2021, Tia/Mana/Alin sira hetan violensia seksual ruma husi familia uma laran? (Peskizador temi violensia abuzu ida-idak iha kraik ba responden). | |
| Halo komentariu seksual kona-ba ita nia isin no roupa ne’ebe ita hatais |  |
| Koalia ho liafuan ruma ne’ebe provoka ita atu halo seksual |  |
| Seluk-seluk tan (bele aumenta husi responde) |  |
| Nunebe Tia/Mana/Alin sira hetan violensia sira ne’e?  Tanba sa mak nalo familia sia halo violensia ne’e sia ba o? |  |
| Oinsa Tia/Mana/Alin hasoru no adapta situasaun sira ne’e ? |  |

**INTERVIEW GUIDE ENGLSIH VERSION**

| **Interview Guide** | |
| --- | --- |
| Sex |  |
| Aged |  |
| Education level |  |
| Occupation |  |
| Address |  |
| Date of Interview |  |
| **Interview Questions and Answers** | |
| **Question 1. Physical violence**  **Did you experience physical violence from your family members during COVID-19 pandemic 2020-2021?** | |
| Probes: How did you get all this violence?  What do you think are the risk factors or underlying reasons for your family members committing such violence against you? |  |
| How did you cope with this situation? |  |
| **Question 2. Verbal violence and Hate Speech (communication violence)**  **Did you experience verbal violence or hate speech (communication violence) from your family members during COVID-19 pandemic 2020-2021?** | |
| How did you get all this violence?  What do you think are the risk factors or underlying reasons for your family members committing such violence against you? |  |
| How did you cope with this situation? |  |
| **Questions 3. Psychology violence**  **Did you experience psychological violence from your family members during COVID-19 pandemic 2020-2021?** | |
| **Probes**: Isolate you from society or confine you to not have contact with outside.  Close all the information to you and do not provide information to you,  Give you misleading information.  Threatening or intimidation  How did you get all these violence?  What do you think are the risk factors or underlying reasons for your family members committing such violence against you?  How did you cope with this situation? |  |
| **Question 4. Sexual violence.**  **Did you experience sexual violence from your family members during COVID-19 pandemic 2020-2021? Probes:** | |
| 1. Forcing you to have sex that you don’t want to do. |  |
| 1. Forcing you to do anal penetration |  |
| 1. Forcing you to do oral penetration |  |
| 1. Other |  |
| Why did you get all these violence? |  |
| How did you cope with this situation? |  |
| **Question 5. Social-Economic Violence**  **Did you experience social-economic violence from your family members during COVID-19 pandemic 2020-2021?** | |
| Taking away or snatching your earnings (income) |  |
| You are not authorized to earn income e.g. just keep you as a housewife. |  |
| Did your husband do any violence that cause you to be disable? |  |
| Did not give you money to manage (if you are a mother) |  |
| How did you get all these violence?  What do you think are the risk factors or underlying reasons for your family members committing such violence against you? |  |
| How did you cope with this situation? |  |
| **Question 6. Domestic violence or intimate partner violence**  **Did you experience domestic violence from your husband during COVID-19 pandemic 2020-2021?** | |
| Physical violence (beating, punching, and kicking) from your husband. |  |
| Physical violence and torture from your husband that cause you to be disable. |  |
| Psychological and economic violence from your husband which caused you wound and hurt. |  |
| Forcing you to have sex even though you don’t want |  |
| How did you get all this violence?  What do you think are the risk factors or underlying reasons for your family members committing such violence against you? |  |
| How did you cope with this situation? |  |
| **Question 7. Violence and Sexual Abuse**  **Did you experience sexual abuse violence from your family members during COVID-19 pandemic 2020-2021? (Interviewers mention each domestic violence below to respondent).** | |
| Giving sensual comment about your body and dress |  |
| Talking some words that provoking for sexual. |  |
| How did you get all this violence?  What do you think are the risk factors or underlying reasons for your family members committing such violence against you? |  |
