# Supplementary material for: A qualitative exploration of the impact of the COVID-19 pandemic on gender-based violence against women living with HIV or tuberculosis in Timor Leste
Source: PLoS One. 2024 Aug 12;19(8):e0306106. doi: 10.1371/journal.pone.0306106 (PMC11318865; doi:10.1371/journal.pone.0306106)
Supplement: Attachment — Submitted filename: Response to Reviewers.docx [file pone.0306106.s002.docx]

**Response to Reviewers**

Dear Editor,

Thank you for considering our paper for publication in **PLOS ONE**. Herewith we submit the revised version of our paper entitled: **“A qualitative exploration of the impact of the COVID-19 pandemic on gender-based violence against women living with HIV or tuberculosis in Timor Leste”**. We have improved the manuscript and addressed each of the comments from the reviewer as reported in the ‘a point-by-point response’ below.

On behalf of all authors,

Sincerely,

Nelsensius Klau Fauk

**Journal Requirements:**

When submitting your revision, we need you to address these additional requirements.

1. Please ensure that your manuscript meets PLOS ONE's style requirements, including those for file naming. The PLOS ONE style templates can be found at

https://journals.plos.org/plosone/s/file?id=wjVg/PLOSOne_formatting_sample_main_body.pdf and

https://journals.plos.org/plosone/s/file?id=ba62/PLOSOne_formatting_sample_title_authors_affiliations.pdf

**Response**

- The manuscript has been adjusted to the journal guidelines.

2. We note that the grant information you provided in the ‘Funding Information’ and ‘Financial Disclosure’ sections do not match.

When you resubmit, please ensure that you provide the correct grant numbers for the awards you received for your study in the ‘Funding Information’ section.

**Response**

- Grant information has been revised, and the number has been provided.

3. Thank you for stating the following financial disclosure:

"Global Fund Timor Leste (East Timor)"

Please state what role the funders took in the study.  If the funders had no role, please state: "The funders had no role in study design, data collection and analysis, decision to publish, or preparation of the manuscript."

If this statement is not correct you must amend it as needed.

Please include this amended Role of Funder statement in your cover letter; we will change the online submission form on your behalf.

**Response**

- "The funders had no role in study design, data collection and analysis, decision to publish, or preparation of the manuscript."

4. Please provide a complete Data Availability Statement in the submission form, ensuring you include all necessary access information or a reason for why you are unable to make your data freely accessible. If your research concerns only data provided within your submission, please write "All data are in the manuscript and/or supporting information files" as your Data Availability Statement.

**Response**

- Data availability statement:

We would like to explain that the data cannot be shared publicly because of restrictions set by the human research ethics committees. Data presented in this study are available on request from the ethics committee imposing the restriction upon the data or from the corresponding author for researchers who meet the criteria for access to confidential data.

Our data in this study comprise transcriptions of digital audio recordings of each individual interview and focus group discussions with women living with HIV or TB in Timor Leste. The interviews were focused on exploring their views and experiences about gender-based violence against them from spouses or family members during the COVID-19 pandemic and the risk factors.

The nature of the restrictions on the data is ethical because participants involved in this study are considered by the ethics committee to be highly vulnerable groups, and data obtained from them may contain sensitive information.

The full name of the ethics committee imposing the restrictions upon the data is the Institute National of Health Research Ethics and Technical Committee. For more information regarding ethical approval of the project please contact the Executive Officer on +67077234849.

**Additional Editor Comments:**

**Introduction and Background**

Weaknesses & Recommendations:

**1. Comment**:

• Lack of Local Context: The introduction mentions global statistics and dynamics of GBV but misses local data critical for contextualizing the study within Timor Leste.

• Actionable Recommendation: The authors should add a paragraph specifically summarizing existing research or reports on GBV, HIV, and TB within Timor Leste. This could be added after the global overview, before transitioning into the study's focus on COVID-19's impact (Introduction, paragraph 3).

• Specific Change: Incorporate statistics from Timor Leste's national health surveys or reports by local NGOs/INGOs that highlight the pre-pandemic situation regarding GBV, HIV, and TB.

**Response:**

- Thanks for your comments, we have added the following paragraph in the introduction section:

“Timor-Leste is a country with significant rates of GBV. Limited evidence suggests that approximately one-third of Timorese women have encountered GBV, which includes physical abuse, coercion, harassment, or being deprived of freedom, while around 59% of Timorese women aged 15-19 have undergone sexual violence at some point in their lives [19, 20]. With the first HIV case reported in 2003, Timor-Leste has registered 1,704 cases, and a significant increase in HIV prevalence has been reported among pregnant women and key population groups [19, 20]. Despite a low overall prevalence of 0.30%, there's a concerning trend toward a broader HIV/AIDS epidemic, extending beyond key populations to the general population [20, 21]. Women living with HIV, along with other vulnerable groups, face challenges in negotiating safer sex and accessing prevention resources [22]. Compounding this issue is the prevalent stigma and discrimination against people living with HIV/AIDS, impacting their lives and access to healthcare [23]. In addition, Timor Leste has the second-highest TB incidence rate in Southeast Asia, 486/10000 population in 2022, with more men than women affected [24]. Factors contributing to TB infections include smoking, alcohol consumption, malnutrition, and poverty [24].”

**2. Comment:**

• Intersections of COVID-19, GBV, HIV, and TB: The introduction broadly links these issues but lacks depth in literature exploration.

• Actionable Recommendation: Deepen the literature review by including studies that have specifically explored the exacerbation of GBV against women with infectious diseases during crises, with a focus on HIV and TB (Introduction, paragraphs 4-5).

• Specific Change: Add references to studies or theoretical models that discuss the vulnerabilities of women living with HIV or TB during pandemics, detailing how societal, economic, and health system pressures during COVID-19 could worsen these vulnerabilities.

**Response**

- Thanks for your comments. We have added the following in the beginning of paragraph 5, including new references:

“There have been associations between the COVID-19 pandemic and violence against women in general in many settings globally [8, 11] and predictions about the possibility of the increase of GBV against women living with HIV during the COVID-19 lockdowns and social distancing [27, 28]. Such predictions are supported by the global evidence suggesting a high prevalence of women living with HIV encountering some form of violence during their lives [29]. Additionally, women with HIV face a disproportionate burden of various inequalities, such as elevated rates of GBV, economic difficulties, lack of access to food, and precarious housing situations [27, 28]. Consequently, there is a specific worry that mandates to 'stay at home' may be unfeasible and hazardous for numerous women living with HIV [27, 28]. Findings from two studies in Brazil and the US suggest the prevalence of 27.8% and 79.6% GBV against women living with HIV in these countries, respectively, during the pandemic [30, 31]. Unemployment status, being diagnosed with post-traumatic stress disorder, the use of illicit drugs, binge drinking, and loneliness have been reported as the associated factors for GBV against them [30, 31]. However, there is a paucity of evidence on the intersections of COVID-19, GBV, HIV, and TB in women or the impact of the COVID-19 pandemic on GBV against women living with HIV or TB and its risk factors in many other settings globally, including in Timor-Leste.”

**Methods**

Weaknesses & Recommendations:

**3. Comment**:

• Snowball Sampling Bias: The methodology briefly mentions the use of snowball sampling but does not address potential biases.

• Actionable Recommendation: Explicitly discuss the potential for network bias and over-representation and describe strategies used to ensure a diverse sample, such as reaching out to different community groups or health centers (Methods, Sampling subsection).

• Specific Change: Add a paragraph discussing the limitations of snowball sampling and mitigation strategies employed, such as efforts to reach beyond initial networks.

**Response**

- We thank you for your comments. We have addressed these comments in the methods section and acknowledged snowballing as a limitation of this study in the limitation section.

“Participants in this study were women living with HIV or TB in eight municipalities (Ainaro, Bobonaro, Covalima, Ermera, Liquiça, Manufahi, Region of Oé-Cusse Ambeno and Dili) in Timor Leste as requested by the Ministry of Health (MOH) and the Global Fund (GF) Timor Leste, that commissioned this project.”

“They were from different community groups in the study settings. The researchers and participants were unknown to each other prior to the study.”

“The current findings should be interpreted with caution to some possible limitations. The use of the snowballing sampling technique and the distribution of the study information sheets through CHCs might have led to the recruitment of participants from the same networks as the current participants and the recruitment of the participants who had been connected to HIV/TB care and treatment. As a consequence, we may have under-sampled women living with HIV or TB who were not on HIV/TB care and treatment and who may have different stories regarding GBV facing them during the pandemic.”

Additional response: “As is the case with many other qualitative studies, this study was not designed to claim generalisability of the findings. However, the richness of the findings or information generated from this study can be useful to inform policy and practice in the study setting and other similar settings in Timor Leste and beyond.”

**4. Comment**:

• Data Analysis Transparency: While the data analysis process is outlined, there are no examples of how themes were developed from data.

• Actionable Recommendation: Provide a detailed example of thematic development, from initial coding to theme generation, possibly in a box or table format to illustrate the process (Methods, Data Analysis subsection).

• Specific Change: Insert a table or text box showing an example of raw data (quotes), initial codes, and how these codes were grouped into themes.

**Response**

- Thanks for your comments. We have described how codes were grouped together to form a theme:

“For example, a range of codes regarding the participants’ (both married and non-married) various experiences of GBV from spouses, family members, and in-laws, including physical (e.g., being beaten, kicked, or slapped), sexual (e.g., forced sexual intercourse), emotional (e.g., being pressured), and verbal (e.g., insults, being scolded or shouted at) abuses were grouped together under the theme: ‘women’s experiences of GBV during COVID-19 pandemic’. Similarly, various codes on how HIV or TB-positive status led to physical and verbal violence against women, and supporting conditions, such as living with other family members in the same house, the dislike of others towards the women’s HIV or TB-positive status were grouped under sub-theme: “*HIV or TB-positive status of women”.* Codes on women's responsibilities in the family and other family members' expectations of them, the position of women or wives in the family and the supporting conditions, such as weak physical condition, feeling tired, and staying at home for an extended period during lockdowns, were classified under the sub-theme: “Women’s responsibility within households”. Codes regarding economic and financial difficulties experienced during the COVID-19 pandemic, loss of job, and financial incapability to pay their children's tuition fees and fulfil necessities, and how these led to women being divorced by their husbands, the sale of properties or personal belongings and spousal disputes, were placed under the sub-theme: “Economic and financial difficulties during COVID-19 pandemic”. The three sub-themes were used to form the theme: “Risk factors for GBV against women living with HIV or TB”. Likewise, various codes explaining individual attitudes and behaviours of male partners, spouses or other family members, such as disapproval of parents, spouses, or other family members about women being late returning home after activities, partners’ or spouses’ jealousy and excessive alcohol drinking, and how these led to spousal dispute, physical, verbal, and psychological violence against women, were grouped under the theme: “Individual-level risk factors for GBV against women during the COVID-19 pandemic”. Finally, codes on how GBV led to psychological impacts on the women, such as emotional repression, heightened stress levels, fear, and feelings of loneliness and isolation, were grouped under the theme: “Psychological impacts of GBV on women living with HIV or TB“;”

- As we have described in the methods section that data analysis and coding were conducted in Tetum language, and only the selected quotes for this publication were translated into English, we are sorry that we are unable to provide the raw data and initial codes.

“Data analysis and coding were conducted in Tetum language to retain the cultural, religious and social meanings attached to the information provided by the women [42]. The selected quotes for this publication were translated into English by NM, MAM, and NKF, who are fluent in Tetum and English.”

**Results**

Weaknesses & Recommendations:

**5. Comment**:

• Link to Research Questions and Objectives: Findings are rich but not explicitly connected back to the study objectives.

• Actionable Recommendation: For each major finding, include a sentence or two that directly ties it back to how it addresses a specific research question or objective outlined in the introduction (Results, start or end of each major finding subsection).

• Specific Change: Add introductory sentences to each subsection of the results that restate the relevant research question or objective the subsection addresses.

**Response**:

- Thanks for your comments. We have provided this at the start of each major theme:

“This section specifically addresses the study objective of exploring the women’s experiences with GBV in different manifestations amid the COVID-19 pandemic, encompassing instances of physical, sexual, verbal, and emotional abuses.”

“This theme addresses the research objective of understanding risk factors for GBV against women during the COVID-19 pandemic. It outlines several main risk factors, such as the HIV or TB-positive status of women, their responsibility within households, economic or financial difficulties, and a spectrum of individual-level risk factors.”

”This section addresses the psychological repercussions of GBV endured by women during the COVID-19 pandemic. These consequences encompassed emotional repression, heightened stress levels, fear, and feelings of loneliness and isolation.”

**6. Comment**:

• Psychological Impacts Underexplored: There's a missed opportunity for deeper analysis on the psychological impacts of GBV.

• Actionable Recommendation: Include a subsection dedicated to exploring the psychological impacts, supported by direct quotes from participants and a more nuanced interpretation of these experiences (Results, create a new subsection on psychological impacts).

• Specific Change: Expand the analysis of participant narratives to include psychological themes, such as feelings of isolation, stigma, or mental health struggles, with direct quotes to illustrate.

**Response.**

- We thank you for your comments. We have added a section (theme) on psychological impacts of GBV:

Psychological impacts of GBV on women living with HIV or TB

This section addresses the psychological repercussions of GBV endured by women during the COVID-19 pandemic. These consequences encompassed emotional repression, heightened stress levels, fear, and feelings of loneliness and isolation. Accounts from women depicted experiences of emotional suppression and a sense of isolation and detachment stemming from the GBV they encountered. The majority felt compelled to hide their emotional anguish, striving to maintain an illusion of normalcy to protect themselves and their loved ones from further harm. For instance, one woman living with HIV described feeling stressed and emotionally stifled but chose to remain silent to avoid stirring up trouble, fearing that her actions might lead to her mother receiving abuse from her husband's family. Similarly, another participant shared her reluctance to disclose her emotional turmoil to her family, opting instead to portray a facade of well-being. This suppression of emotions was also echoed by another TB patient who felt obliged to endure her situation silently because of her role as a married woman and an outsider in her husband's community:

*“I was stressed out and felt emotionally suppressed due to the abuses, but I was scared, just quiet and didn’t make any problem because my mum could also get beaten by my husband's family. I felt lonely and isolated in this situation” (Interview, participants with HIV).*

*“I never tell my family and others about what I have been going through, emotional pain I experience. I pretend to show to them that I’m fine” (Interview, participants with TB).*

*"I am a married woman, and I should be patient. And I am only a visitor in my husband's hometown, so I can’t go against them. I feel the pressure emotionally due to my husband’s harsh attitudes and behaviours, but I couldn't do anything” (Interview, participants with TB).*

For some women, the experience of gender-based violence they faced also led to feelings of isolation and alienation due to the inability to confide in others or the absence of supportive individuals. The feelings of loneliness and detachment were stark in the narratives of these women. One woman living with HIV expressed a profound sense of isolation, exacerbated by the absence of someone with whom she could share the emotional burden she faced. This isolation was intensified by the tragic loss of her child, a devastating event met with indifference from her husband's family. Similarly, another woman living with HIV who endured GBV from her husband and in-laws shared a heart-wrenching account of feeling that her world had become empty and dark due to the overwhelming stress and pressure, compounded by a lack of support networks, particularly after the loss of her child. The lack of care and concern from her husband's family during such a traumatic experience accentuated her suffering and deepened her sense of alienation:

*“They [husband’s family] insulted me many times and didn’t care at all about me and my kids. And even when one of my kids passed away, they didn’t come to visit us. My family also never visited me either. I have no one to share the emotional pain I feel****”*** *(Interview, participants with HIV).*

*"I was insulted, abused by my husband and his family. I felt all the stress and pain. I felt that everything was empty to me and dark, and even more when I lost my child, and a family from my husband's side did not care about me, I was so suffering”* *(Interview, participants with HIV).*

**Discussion and Conclusions**

Weaknesses & Recommendations:

**7. Comment**:

• Policy and Practical Implications: The discussion highlights findings within broader literature but lacks depth on implications.

• Actionable Recommendation: Expand the discussion on policy implications by suggesting specific policy changes or interventions needed in Timor Leste based on the findings, such as integrating GBV prevention strategies into existing HIV/TB programs (Discussion, Implications subsection).

• Specific Change: Add paragraphs that detail specific policy recommendations, including programmatic changes or new interventions targeting the identified risk factors.

**Response:**

- This is great recommendation; indeed, policy and programmatic recommendation are important. We have added the recommendations as suggested:

Recognising and addressing the gendered impacts of the pandemic in Timor Leste is crucial. The pandemic has exacerbated existing societal inequalities, making women, especially those with HIV or TB, more vulnerable to GBV. Policies in Timor Leste should be specifically designed to support these women, providing not only protection against GBV but also acknowledging the cultural nuances. Challenging and transforming traditional gender roles and expectations, deeply rooted in Timorese society, is vital. These societal norms significantly contribute to GBV, particularly in times of crisis. Interventions aimed at modifying these perceptions and promoting gender equality can create a safer and more equitable environment for women [51].

It is evident that the disease causes financial stress in the family, which is intensified by the pandemic. Disease causes temporal disfunction of the women, it creates dependency which is a significant factor that increase the vulnerability of women to GBV. Providing financial empowerment and support initiatives can play a crucial role in mitigating these risks and relieving the pressure on the survival of GBV. Additionally, addressing individual-level risk factors, such as jealousy and alcohol consumption among male partners, is essential. Enhancing awareness about GBV in Timor Leste and developing accessible reporting mechanisms, self-reporting, massive media campaigns, and scaling up existing safe space for women's survival of GBV are essential. These methods can assist women in safely reporting incidents of GBV [52]. Lastly, strengthening collaboration between the government, NGOs and civil society organisations is key to developing and implementing comprehensive GBV policies. Such partnerships should ensure dedicated resources and responses specifically tailored to the challenges posed by the pandemic in Timor Leste. This approach can enhance the effectiveness of GBV interventions and provide a framework for ongoing support and protection of women, particularly in the post-pandemic period [51-53]

• Generic Recommendations for Future Research: The suggestions for future research are broad and not specific.

• Actionable Recommendation: Specify areas where future research is critically needed, perhaps identifying specific populations, geographic areas, or thematic gaps that the current study has uncovered (Discussion, Future Research subsection).

• Specific Change: Provide a list of potential research questions or methodologies that future studies could adopt, based on the gaps identified in the current research.

**Responses:**

- A recommendation for future studies has been added:

“There is a need to employ mixed-methods research to gain a comprehensive understanding of the issue. Qualitative approaches can provide in-depth insights into the personal experiences of GBV, while quantitative methods can offer broader epidemiological perspectives, in the other hand exploring the existing policy and strategy implementation will also generate evidence on the policy and strategic gaps.”

**Overall Scientific Soundness and Originality**

Recommendations for Improvement:

• Methodological Rigor: Detail the analytic process more thoroughly to ensure findings are grounded in data.

• Specific Change: Add an appendix or supplementary material that outlines the coding framework or provides examples of the coding process, to enhance methodological transparency.

- See response to comment No. 4

• Enhance Originality: Draw clearer connections between findings and their implications for Timor Leste's specific context.

• Specific Change: Incorporate a section discussing how the study's findings contribute new insights specific to Timor Leste and how these insights could influence local practice or policy differently from other contexts.

- See response to comment No. 7

**Response**:

- Thanks for your comments, we believed we have addressed the comments throughput the manuscript and the details have been provided in responding to the above comments 1-7.

**Quality of English Language**

**Comment**:

• Specific Change: Engage a professional editing service familiar with academic writing in the public health field to ensure clarity and adherence to academic standards.

**Response**:

- We have edited the language throughout the manuscript.

**Review Comments to the Author**

Reviewer #1: Please, please include page and line numbering in your submissions. It is almost impossible to indicate where comments should go otherwise!

**Introduction**

**Comment**:

‘using COVID-19 restrictions and the threat of the infection [10, 16].’ What does ‘threat of the infection’ mean? Do you mean they threatened to inform others of the infection?

‘Being younger and having more children and adolescents were also significantly associated with GBV against women during the COVID-19 lockdown [13].’ Do you mean having adolescent children or being an adolescent? ‘Timor Leste’s referral system includes three levels…across the territory [31].’ Is this relevant in an already quite long paper?

**Response:**

- Thanks for your comments, we have made necessary revisions.

**Results**

**Comment**:

Here you mention that many participants were illiterate, but you have not discussed this in the consent or ethics sections. How did you deal with illiteracy when signing consent forms that are by definition written? Did you have witnesses to the explanation of the study? How many participants were illiterate? I would suggest that this might be a key variable in your analysis but does not seem to be covered in the results.

**Response**:

- Thanks for your comment, we have addressed it in the “Ethical Consideration” section:

“Before commencing the interviews, the participants were given a chance to read a written informed consent and sign it. For the participants who could not read, the field researchers read the content of the informed consent for them. All participants were given a chance to raise questions or ask for clarification after reading or listening to the informed consent. They were also informed during the recruitment that they were allowed to bring somebody they trusted to be with them during the interviews or FGDs, but none of them did so.”

**Comment**:

Did women reporting violence also report violent behaviour pre-pandemic? This would be interesting to know. I am struggling to see the connection explicitly (I know, men were at home more etc etc, but I think this needs to be clarified).

For example: ‘During the COVID pandemic, I felt sad and worried about my neighbours because they sometimes insulted me by asking me how come my family and I had TB and HIV. It was a big concern for me because I am a [university] student, and I am still afraid that my friends will stay away from me and hate me due to the disease that I have.”’ Okay, but how is this related to/worsened by Covid?

‘My husband worked at a store in Dili [capital city of Timor Leste], but because of the

COVID-19 outbreak, my husband lost this job and only stayed at home. I was not working either, just looking after my children. We had no money, …. When my husband was angry, he would beat and swear at me. He abused me. I think he did that because he was stressed and felt pressured. Because COVID-19 came, our products were not sold, and there was also no money, which caused us problems. I was stressed because of no money, my ill-health condition and his violent attitudes and behaviours” (FGD, participant with TB)’

**Response**:

- Thanks for your comments, we have added the following to make it clear:

“The latter participant further explained that she had not encountered such a cynical and harsh question before the pandemic because she used to attend classes on campus daily and didn't frequently interact with neighbours: "*Before COVID, I seldom conversed with them [neighbours] because I spent most weekdays on campus attending classes*." She highlighted that neighbours within her communities interacted more often during the lockdowns: *"It seemed that due to the lockdowns, neighbours connected or encountered each other more frequently than before COVID*."

“The above women's narrative showed that she had not encountered such experience prior to the pandemic: "*He [her husband] wasn't used to being angry like this before COVID. Besides, we only saw each other on weekends because he worked in the city*."

**Comment**:

Again, this quote suggests there was little difference financially pre- and during Covid, except that her husband was at home.

There is some repetition in quotes, e.g.,

‘I got so many insults from my brother and sister-in-law; they kicked me out of the home” (Interview, participant with HIV).’

Next page: ‘I got so many insults from my brother and sister-in-law. They kicked me out of home. They told me that I had a bad illness and that I should stay away from them. ….. They often yelled and screamed at me when I stayed close to them. During the COVID-19 lockdowns, we were at home most of the time; I couldn’t stay away from them because we lived in the same house. That is why the insults I got from them intensified during those lockdowns. …. They talked badly and swore me many times” (Interview, participant with HIV).’

There is generally quite a lot of repetition in this section, and maybe too many illustrative quotes used to make the same point again.

**Response**

- We thank you for your detailed observation. We have deleted the first one.

**Comment**:

The individual level risk factors section is probably the most interesting part of the results, but is the shortest. If you have any more material for this section I would include it and cut down some of the earlier sections.

**Response:**

Thanks for your important suggestion. Unfortunately, those presented under individual level risk factors are the ones we have. We have recommended individual level risk factors to be explored more in future large-scale studies.

*Reviewer* #2: I think this manuscript is well researched and written, good job! Minor comments:

**Comment**:

1) Interviews and focus groups were conducted in Tetum. Who translated them to English and what was the process?

**Response**

- Thanks for your comments, we have provided this in the data nalaysis section:

“Data analysis and coding were conducted in Tetum language to retain the cultural, religious and social meanings attached to the information provided by the women [42]. The selected quotes for this publication were translated into English by NM, MAM, and NKF, who are fluent in Tetum and English. To maintain the accuracy of the translation and the interpretations, other authors, who speak both languages, checked and re-checked both versions [43].”

**Comment**:

2) Why did you choose to sample HIV and TB women? What is the motivation? What are their existing risk factors? I know COVID worsened it, especially in the area of GBV. However, were they already experiencing GBV before covid? How does the GBV they experience compare to the rest of the population in TL? This is a missing piece of the puzzle to address. I think it is important to highlight how covid made GBV especially worse for this population, and if it really is the case, why does HIV/TB make life so difficult for these women?

**Response**

- Thank you for these important questions.

Firstly, we must acknowledge that to the best of our knowledge, no official report or research is reporting specifically about GBV in women with HIV OR TB or the general population in Timor Leste. Although some evidence suggests that “approximately one-third of Timorese women have encountered domestic violence, which includes physical abuse, coercion, harassment, or being deprived of freedom, while around 59% of Timorese women aged 15-19 have undergone sexual violence at some point in their lives [19, 20]”, there is a lack of evidence on GBV and risk factors for GBV against women and girls in the country. Thus, this is an initial study that can inform further exploration of this issue through large-scale quantitative study designs to understand the magnitude of the issue and drivers/risk factors for GBV against women and girls in the country.

The specific focus on women living with HIV or TB, as described somewhere in the method section, was based on the request from the Ministry of Health and the Global Fund (GF) Timor Leste to establish evidence on this issue, which can lead to policies and interventions aiming at supporting these vulnerable groups in Timor Leste.

- We have also provided some evidence on the intersections of COVID-19, GBV, HIV, and TB among women in the introduction section to justify the focus of this study:

“There have been associations between the COVID-19 pandemic and violence against women in general in many settings globally [8, 11] and predictions about the possibility of the increase of GBV against women living with HIV during the COVID-19 lockdowns and social distancing [27, 28]. Such predictions are supported by the global evidence suggesting a high prevalence of women living with HIV encountering some form of violence during their lives [29]. Additionally, women with HIV face a disproportionate burden of various inequalities, such as elevated rates of GBV, economic difficulties, lack of access to food, and precarious housing situations [27, 28]. Consequently, there is a specific worry that mandates to 'stay at home' may be unfeasible and hazardous for numerous women living with HIV [27, 28]. Findings from two studies in Brazil and the US suggest the prevalence of 27.8% and 79.6% GBV against women living with HIV in these countries, respectively, during the pandemic [30, 31]. Unemployment status, being diagnosed with post-traumatic stress disorder, the use of illicit drugs, binge drinking, and loneliness have been reported as the associated factors for GBV against them [30, 31]. However, there is a paucity of evidence on the intersections of COVID-19, GBV, HIV, and TB in women or the impact of the COVID-19 pandemic on GBV against women living with HIV or TB and its risk factors in many other settings globally, including in Timor-Leste.”
